# Apnoeic oxygenation with high flow nasal oxygen for interventional surgery of the larynx and pharynx

**DOI:** 10.1007/s00405-024-08726-6

**Published:** 2024-05-16

**Authors:** Christine Langer, Claus Wittekindt, Christoph Arens, Sonja Käbisch

**Affiliations:** 1 Klinik für HNO-Heilkunde, Kopf-/Halschirurgie, Plastische Operationen, Universitätsklinikum Gießen und Marburg, Justus-Liebig-Universität Gießen, Standort GießenKlinikstraße 33, D- 35392 Giessen, Germany; 2 Klinikum Dortmund, Klinik für HNO-Heilkunde, Dortmund, Germany; 3 Klinik für Anästhesie, Universitätsklinikum Gießen und Marburg, Standort Gießen, Giessen, Germany

**Keywords:** HFNO, THRIVE, Larynx surgery, Highflow oxygenation, Apnoeic ventilation

## Abstract

**Background:**

Highflow nasal cannula oxygen (HFNO) is known to be used for noninvasive oxygenation in intensive care patients but it has rarely been used in airway management for elective surgery of the upper aerodigestive tract.

**Objectives:**

HFNO offers opportunities of a tubeless oxygenation system which is easy to handle and not limited only on surgery of the endolarynx.

**Methods:**

We evaluated this method for oxygenation during brief interventional procedures of the larynx and pharynx in 92 adult patients for safety and intraoperative complications. The need of secondary endotracheal intubation and limiting comorbidities as pulmonal and cardiac diseases were documented.

**Results:**

HFNO showed a good safety profile concerning saturation and hypercapnia. Oxygen desaturation below 90% occurred only in 5 patients, mask ventilation led to quick recovery except in one patient who was secondary intubated. A significant influence of the body mass index on the minimal O2 saturation was shown (p < 0,001) so that a possible limitation of the method exists here. Comorbidities were grouped into the ASA classification. There was a significant difference between ASA I/II and ASA III patients in terms of minimum O2saturation.

**Conclusion:**

We conclude that HFNO may hold great promise for changing ventilator technique in general anesthesia, particularly in short elective laryngeal and pharyngeal surgery. Safety and feasibility were proven in this study.

## Introduction and objectives

High-flow nasal cannula oxygen (HFNO) was primarily developed for neonatal intensive care. Over the years it found additional application in adults, mostly in intensive care units but also in severe trauma and other critically ill patients. Today it is routinely applied in adult intensive care in severe pulmonal (Acute Respiratory Distress Syndrome (ARDS), COVID 19) as well as in cardiac disorders [1, 2].

HFNO is easily set up and delivers a 100% oxygen flow (up to 70 l/min), warmed and humidified. Compared to Non-Invasive Ventilation (NIV) masks, patient´s comfort is improved, ciliary dysfunction, infection and damage to airway mucosa is limited. HFNO provides both flushing of dead space and positive airway pressure, thus preventing airway collapse and atelectasis. Continuous insufflation facilitates oxygenation and carbon dioxide clearance through gaseous mixing and deadspace washout [3, 4]. Airway patency is crucial and must be maintained throughout use of HFNO.

The first indications for use in adult patients were severe pulmonary (ARDS) and cardiac disorders [5, 6]. HFNO was primarily applied in postoperative cardiac surgery patients with extubation failure [7]. Compared to conventional NIV therapy HFNO proved to be equivalent regarding mortality and re-intubation rates.

HFNO has been used in airway instrumentation during elective or urgent airway manipulations such as bronchoscopy [8], but also to improve the safety of airway management during apnea before intubation (difficult airway, bariatric surgery, obesity) [9].

HFNO has rarely been used in airway management for elective surgery of the upper aerodigestive tract [10, 11]. Performing suspension microlaryngoscopy or pharyngoscopy requires sufficient depth of anesthesia to tolerate rigid instruments and their suspension. To perform suspension microlaryngoscopy or rigid pharyngoscopy, different anesthesiological techniques are used, each with different advantages and disadvantages. After neuromuscular blockade patient is orally intubated to control airway and endotracheal ventilation. The disadvantage of this technique is an obstruction of parts of the surgical site by the endotracheal tube. To solve this surgical problem, tubeless oxygenation systems as jet ventilation were developed in the 1990’s [12] and are still the standard of care for performing laryngeal surgery in an unobstructed airway. Jet ventilation requires special equipment as well as an experienced team of surgeons and anesthesiologists. Furthermore, surgery is anatomically reduced to the glottic area as jet ventilation needs an optimal exposure of the airway.

In the following study, the use of HFNO during short time planned surgical interventions of larynx and pharynx regarding intraoperative complications was investigated, need of secondary endotracheal intubation and limiting comorbidities as pulmonal and cardiac diseases were documented. The aim of the study was to investigate whether endotracheal intubation or jet ventilation can be easily and safely replaced by continuous HFNO apnoeic oxygenation.

## Methods

Between 1/2018 and 1/2020 we performed non-laser pharyngeal and laryngeal surgeries in 93 adult patients with benign and malign lesions of the upper aerodigestive tract. Patients had to be at least 18 years old and able to understand and consent to the study conditions. Patients with a planned selective procedure on the upper aerodigestive tract (larynx, oropharynx, hypopharynx) were included. Inclusion criteria were a planned surgery time up to 20 min and a low probability of severe bleeding during the operation. Furthermore, severe gastro-esophageal reflux was a contraindication; patients with mild or sufficiently treated reflux were included. Patients with critical airway obstruction were excluded from participation in the study, as were patients with a bleeding tendency or coagulation disorders.

**Fig. 1:**
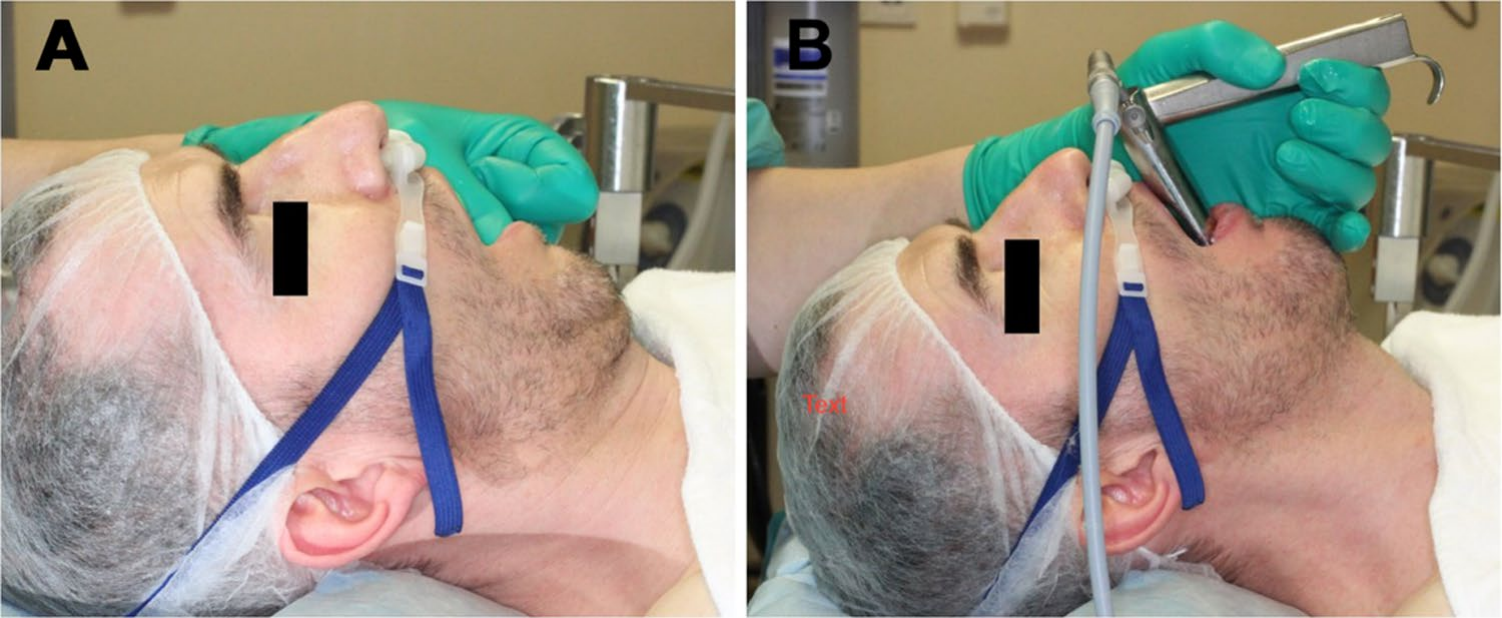
Intraoperative setting of HFNO suspension laryngoscopy, **A** position of the nasal prongs (Opti Flow^©^) during preoxygenation, **B** insertion of the suspension laryngoscope with persisting nasal prongs

Epidemiological data concerning age, sex, nicotine consumption, comorbidities, indication for surgery, intra- and postoperative complications and the need of airway conversion, e.g. endotracheal intubation during surgery, were documented. All patients were preoxygenated with the Opti Flow nasal prongs (Opti Flow, Fisher & Paykel Healthcare Ltd.), beginning at a rate of 20 l/min up to 70 l/min as patients lost consciousness (Fig. 1). Intravenous anesthesia induction started with Fentanyl boluses (1–5 µg/kg), Propofol (2–3 mg/kg) and Mivacurium (0,14–0,25 mg/kg) followed by peripheral continuous infusion of Remifentanil (0,2–0,5 $\mu$ g/kg/min) and Propofol (0,1–0,2 mg/kg/min). When patients lost consciousness, mask ventilation was confirmed and discontinued. Measurement of apnea time began at this moment. Afterwards the suspension laryngoscope was inserted (Fig. 1), and surgery was performed under apnea with continuous HFNO at 70 l/min. At the end of the operation, mask ventilation was resumed, and end-tidal CO_2_ measured until the patient emerged.

Intraoperative monitoring followed AAGBI-guidelines. During the operation, continuous pulse oximetry was performed, and lowest value of oxygen saturation was measured.

The intraoperative setting is shown in Fig. 1 (Fig. 1)*.*

Data were collected as part of standard patient documentation. Statistics were performed using IBM^®^ SPSS^®^ Statistics version 27.0.0.0.

Linear regression was performed to analyze correlation between continuous variables. To compare independent groups of continuous outcomes one-way analysis of variance (ANOVA) was performed. In case of significant differences Tukey’s post-hoc test was used to analyze the dependencies. All statistical tests and confidence intervals were performed on a two-sided 5%-level.

The patients have been informed about participation in the study and have given their written informed consent. The study was approved by local ethics committee of the University of Giessen (AZ 101/22).

## Results

### Epidemiological data

The case series included 93 patients, 49 men and 44 women. Mean age in the entire cohort was 58,6 years, ranging from 20 to 84 years. Indication for surgery was a benign lesion of the larynx in 72 cases, carcinoma or premalignant lesion of the larynx in 13 cases and benign and malignant lesions of the pharynx in 9 cases. Body mass index (BMI) varied from 18,7 to 43,2 kg/m^2^ (mean 27,0 ± 4,7). Nicotine abuse was identified in 62 patients (66,7%) with a mean amount of 29,7 packyears. The preoperatively categorized patient status according to American Association of Anesthesiologists (ASA) ranged from I-III (ASA I / II / III analogous 5/55/33).

### Respiration parameters

The median apnea time was 8,08 min (1–23 min). Minimum oxygen saturation (SpO_2min_) during HFNO averaged 95,5 ± 3,5%. The mean maximum end tidal (et) CO_2_ level (CO_2max)_ was 50,5 ± 7,6 mm Hg.

**Fig. 2:**
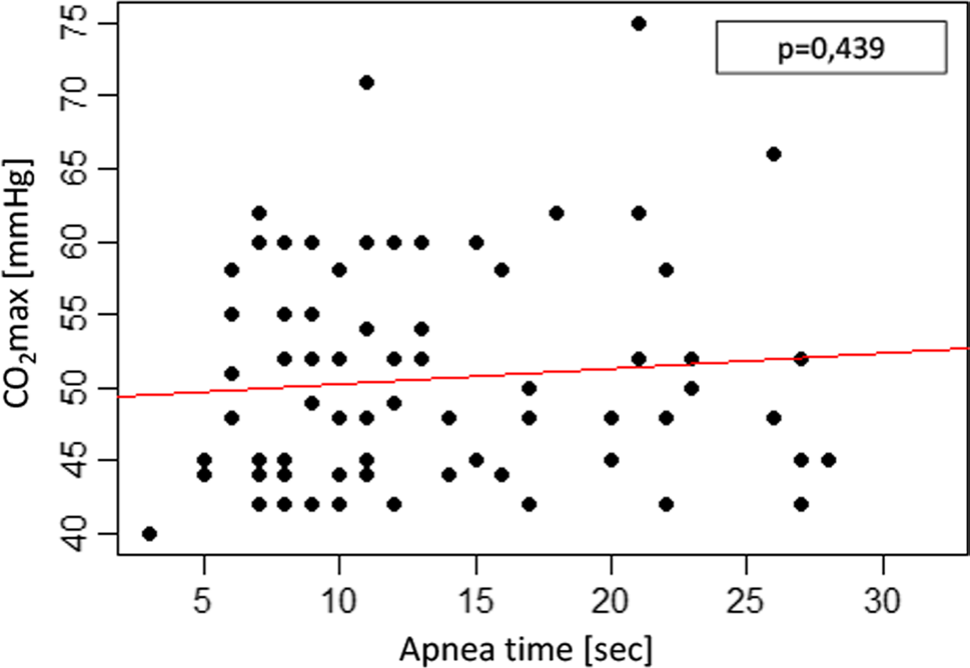
Patient’s maximum end tidal (et) CO_2_ level [mmHg] during intraoperative administration of HNFO correlated with apnea time [sec]

We found no significant correlation of apnea time with end tidal CO_2_ (p = 0.439) as shown in Fig. 2 (Fig. 2).

Patients’ baseline characteristics are shown in Table 1.

**Table 1:** Patients’ baseline characteristics

	Total (n = 93)
Age [years], mean (SD)	58,6 (14,7)
BMI [kg/m^2^], mean (SD)	27,0 (4,7)
ASA physical status, n (%)	
1	5 (5,4)
2	55 (59,1)
3	33 (35,5)
Smoking, n (%)	
Yes	62 (66,7)
No	31 (33,3)
Diagnosis, n (%)	
Benign lesion larynx	71 (76,3)
Malignant lesion larynx	13 (14,0)
Oropharyngeal lesion	9 (9,7)
SpO_2min_ [%],	
Mean (SD)	95,52 (3,47)
CO_2max_ [mmHg],	
Mean (SD)	50,51 (7,59)
SpO_2min_, n (%)	
< 90%	5 (5,38)
≥ 90%	88 (94,62)

Oxygen desaturation below 90% occurred in the following five patients:

#### Patient 1

A 42-year-old male with chronic laryngitis desaturated to 80% under High Flow, end tidal CO_2_ raising up to 71 mmHg. He recovered immediately after mask ventilation, we intubated the patient to complete surgery. His BMI was 35,8 kg/m^2^ and he had a history of nicotine abuse and chronic obstructive pulmonary disease (COPD).

In the following four cases desaturation occurred at the end of the surgical procedure, patients recovered quickly after mask ventilation; therefore, intubation was not necessary.

#### Patient 2

A 41-year-old female with subglottic stenosis and a BMI of 40 kg/m^2^ desaturated to 89%, et CO_2_ rising to 58 after six minutes HFNO. She recovered after mask ventilation.

#### Patient 3

A 71-year-old male suffering from glottic carcinoma (BMI 29,4 kg/m^2^, nicotine abuse) desaturated after 6 min apnea to 88%, endtidal CO_2_ measured 42 mmHg. Mask ventilation led to an immediate recovery.

#### Patient 4

A 64-year-old male with oropharyngeal carcinoma (COPD, nicotine abuse, BMI 29,1 kg/m^2^) desaturated to SpO_2_ 85%, endtidal CO_2_ rising to 55 mmHg after only 3 min of apnea. Mask ventilation normalized the measured values immediately.

#### Patient 5

A 62-year-old male with glottic carcinoma and history of nicotine abuse (BMI 26,7kg/m^2^) desaturated to 88%, endtidal CO_2_ climbing to 60mmHg after 8 min of apnea. He recovered immediately after mask ventilation.

### Influence of smoking

As smoking may be a factor of interest according to respiratory parameters, we conducted a one-way analysis of variance (ANOVA) which revealed no significant effect of smoking on CO_2max_ and SpO_2min_. Both missed significance level (CO_2max:_ 95% CI [− 0,777; 6,332] p = 0.124, SpO_2min_: 95% CI [− 2,413; 0,478], p = 0.187).

### Influence of body weight/BMI

**Fig. 3:**
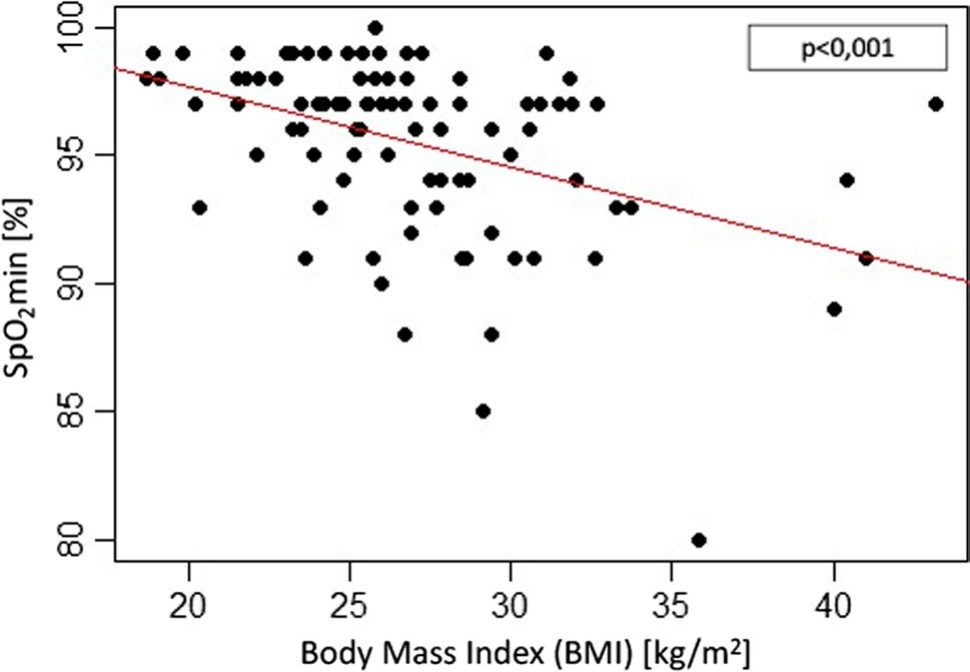
Patient’s lowest oxygenation (minimum SpO_2_) during intraoperative administration of HNFO as measured by pulse oximetry correlated with body mass index (BMI [kg/m^2^])

Body weight was recorded based on BMI. Correlation with end expiratory CO_2_-levels was not significant (p = 0.488), while O_2_-saturation was highly significantly dependent on BMI (p < 0,001) (Fig. 3).

### Influence of age

Neither end tidal CO_2_-levels (p=0,97) nor minimum O_2_-saturation levels (p=0,77) were significantly influenced by patient age.

### Comorbidities and ASA physical status

Patient’s comorbidities were differentiated into cardiovascular (such as hypertension (n = 44), coronary heart disease (n = 7), condition after stroke (n = 3) or ventricular arrhythmia Lown IV (n = 1) and pulmonary diseases (especially COPD Gold I-III (n = 11), bronchial asthma (n = 9)). We found 55 patients with cardiovascular comorbidities and 20 patients with pulmonary diseases. Three patients suffered from obstructive sleep apnea, two of them regularly using a CPAP mask at night. These patients´ oxygen saturation was monitored 24 h after surgery at our ICU. None of these patients showed desaturation through or after the surgical procedure.

**Fig. 4:**
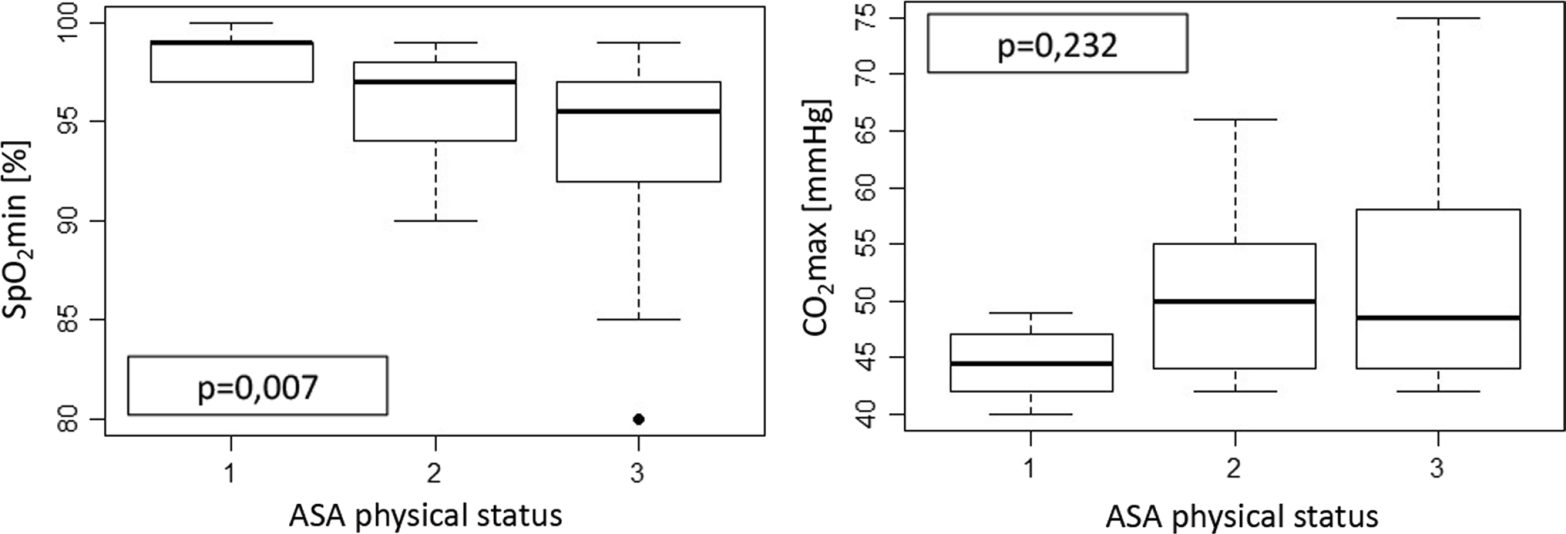
Patient’s lowest oxygenation (minimum SpO_2_) during intraoperative administration of HFNO as measured by pulse oximetry and expiratory level of CO_2_ after end of apnea time correlated with ASA physical status

Considering ASA classification as a measure of comorbidities, we conducted a one-way analysis of variance (ANOVA) to compare ASA physical status with SpO2_min_ and expiratory CO_2_-levels. While ASA performance status had no influence on CO_2_-levels (F-statistics 1,488; p = 0,232), the one-way ANOVA revealed a significant effect with O_2_-saturation (F-statistics 5,242; p = 0,007). Tukey´s post-hoc analysis (95% family-wise confidence level (CI)) revealed a significant difference between scores of the groups with ASA 1 and ASA 3 patients (95% CI [-0,787; -0,319], p = 0.03) and between ASA 2 and ASA 3 patients (95% CI [-3,543; -0,091], p = 0.04) but missed significance level between ASA 1 and ASA 2 patients (95% CI [− 5,962; 1,407], p = 0,31) (Fig. 4).

## Discussion

Airway management in surgery of larynx and pharynx is challenging, especially since the anatomic regions are shared by surgeon and anesthesiologist [12].

Endotracheal intubation leads to a restricted surgical field, which is why alternative tubeless airway management methods such as jet ventilation have been developed, technically optimized and well established for more than 30 years [12–14]. As jet ventilation requires a skilled team and is limited to indications within endolarynx to optimize oxygenation during surgery, nasal high flow oxygenation appears to be an alternative method of airway management. There are numerous studies on the use of jet ventilation in laryngeal surgery, mostly case series, which show complications such as hypercapnia, hypoxia and barotrauma [15–17]. Effective application of JET presupposes specific knowledge and training. Jet ventilation also requires a great deal of equipment. In suspension laryngoscopy, supraglottic ventilation is generally used with suitable suspension laryngoscopes for jet ventilation. The instruments for this are expensive to purchase and maintain. In contrast, the equipment required for THRIVE is significantly less complex and less cost-intensive and the complexity of implementation is low [18]. We therefore see this technique as a high-potential alternative to jet ventilation, which always requires a specialized center to perform [19].

In the present study, a large group of unselected patients undergoing short time laryngeal and pharyngeal surgery both for malignant and benign lesions to investigate feasibility and safety of HFNO was examined. The measurements were integrated into the daily surgical routine. In 92 patients just one case of critical desaturation was observed requiring endotracheal intubation. Four patients with mild desaturation at the end of surgery recovered immediately under mask ventilation. In all four cases the patients had upper airway constriction by tumor or stenosis of larynx or pharynx and therefore patency of the airway maybe was not sufficient.

The data collected in this study can further help to show limitations of this ventilation modality. A critical point of apnea oxygenation with HFNO may be hypercapnia, especially in prolonged surgical interventions [10]. The mechanism and amount of CO_2_-washout in HFNO and the frequency of CO_2_ accumulation with consecutive respiratory acidosis remain undefined.

Compared to low-flow apnea oxygenation carbon dioxide clearance is better in high-flow settings [20, 21]. Studies using either capnography or blood gas analysis showed only moderate rise of pCO_2_ in the first 15 min of surgery [20, 22]. In our study end tidal CO_2_ was measured and showed an uncritical rise of CO_2_ in the majority of patients. If high-flow is used for longer procedures, CO_2_ monitoring beyond capnography is required to monitor ventilation. Blood gas analysis or transcutaneous CO_2_ monitoring are practical ways to obtain this information.

The efficiency of apnea oxygenation is affected by the ratio of functional residual capacity (FRC) to bodyweight. Obese patients may therefore desaturate quickly, and a higher BMI (> 35kg/m^2^) should exclude patients from this technique, especially if the planned surgery time is exceeding 15 min. Previous studies have demonstrated that HFNO is also a safe tool for an induction as well as the postoperative period in morbidly obese patients to improve oxygenation [23, 24]. Recognizing this critical point, four patients with BMI over 40 kg/m2 were included in our study group for short surgical interventions and none of them desaturated under SpO2 of 90%. Nevertheless, our study showed a direct and statistically significant influence of BMI on SpO_2_.

From the surgeon’s point of view, HFNO means to have an unimpeded view to the surgical field. Compared to jet ventilation HFNO is easier to handle, and the operation site is not limited to the larynx. Short surgical procedures within the oropharynx and hypopharynx could be performed safely with HFNO. Oxygenation was also easily possible without any major complications.

Airway patency is crucial when using high-flow technique as well as during jet ventilation. Airway obstruction may lead to faster desaturation as described above.

## Conclusion

HFNO prolongs the safe apnea time and has already changed and facilitated the management of the difficult airway. HFNO may also hold a great promise of transforming ventilation technique under general anesthesia, particularly for laryngeal surgery. Free access (without endotracheal tube) improves surgical precision and may reduce operating time as well as patient´s recovery time.

We conclude that HFNO is an adequate and safe ventilation method for short to mediate long laryngeal and potentially also pharyngeal surgery. Procedures lasting longer than 15 min require close ventilation monitoring. If continuous blood gas analysis or transcutaneous CO_2_ measurement is available, longer procedures (up to 60 min) may be possible.

## Data Availability

The data that support the findings of this study are available on request from the corresponding author.
